# Severe primary organ-related complications in patients with de novo metastatic cancer: implications for prophylactic local therapy

**DOI:** 10.1016/j.ctro.2026.101238

**Published:** 2026-07-17

**Authors:** Hefei Liu, Dafna Merhav, Naia Tabakovic, Ronac Mamtani, Karishma Khullar, John Christodouleas

**Affiliations:** a Department of Radiation Oncology, University of Pennsylvania, USA; b Department of Medical Oncology, University of Pennsylvania, USA; c Elekta, Stockholm, Sweden

**Keywords:** Metastatic cancer, Organ-related complications, Cumulative incidence, Prophylactic radiotherapy, Pancreas, Bladder, Prostate

## Abstract

**Purpose:**

Patients with de novo metastatic cancer develop local organ-related complications leading to hospitalizations, procedures, and reduced quality of life. Prophylactic local therapy (e.g., radiotherapy) has been proposed to reduce morbidity, but the incidence and timing of severe primary organ-related events (SPOREs) remain poorly defined. We quantified the cumulative incidence and spectrum of SPOREs to inform trials of prophylactic local therapy.

**Methods:**

We conducted a retrospective cohort study of 358 patients with de novo metastatic prostate, bladder, and pancreatic cancers treated between 2010 and 2023. SPOREs were defined using clinically meaningful, organ-specific criteria, excluding events within 30 days of diagnosis. Death was treated as a competing event, and cumulative incidence functions were estimated with the Fine–Gray method.

**Results:**

A total of 104 prostate, 59 bladder, and 195 pancreatic cancer patients (adenocarcinoma *n* = 178; neuroendocrine n = 17; including head and body tumors) met inclusion criteria. At 24 months, cumulative incidence of SPOREs was 3.3%, 27.2%, 40.0%, and 45.5%, respectively (*p* < 0.001). Overall survival differed across disease sites but did not parallel SPORE risk. SPORE incidence plateaued after 24 months for bladder and pancreatic adenocarcinoma, while prostate cancer and pancreatic neuroendocrine tumors continued to increase. Urinary obstruction predominated in prostate and bladder cancer, whereas biliary obstruction or cholangitis was most common in pancreatic cancer. Within pancreatic tumors, there was a trend toward higher SPORE incidence for head versus body tumors (48.5% vs 32.7% at 24 months, *p* = 0.054).

**Conclusion:**

SPOREs represent a substantial source of morbidity in de novo metastatic cancer. Across disease sites, risk and timing appear driven significantly by anatomic vulnerability of the primary tumor rather than metastatic virulence or prognosis. Despite differing biology, pancreatic adenocarcinoma and neuroendocrine tumors demonstrated high local morbidity. These findings suggest anatomic context may better identify patients most likely to benefit from prophylactic local therapy aimed at reducing morbidity.

## Highlights

• SPORE risk varies widely across metastatic cancer types.
• SPORE risk does not parallel overall survival across cancers.
• Local morbidity appears to be driven by primary tumor anatomy.
• Pancreatic head tumors show higher SPORE risk than body tumors.
• Findings support trials of prophylactic local therapy.

## 1 Introduction

Patients presenting with de novo metastatic disease may develop significant morbidity from an uncontrolled primary tumor despite the presence of distant disease. Depending on the organ of origin, local progression can lead to emergency interventions, hospitalizations, unplanned procedures, and significant declines in quality-of-life. [1] Investigators have hypothesized that prophylactic local therapy, such as radiotherapy to an intact primary site, may reduce risks related to progression of the primary tumors and overall improve a patient's quality of life, even in the absence of an overall survival (OS) benefit. [2], [3] However, the epidemiology and absolute risks of severe primary organ-related events (SPOREs) in metastatic disease are poorly characterized. In particular, few studies systematically quantify how frequently they occur relative to death. Robust estimates of the incidence of these events are essential to properly design clinical trials of prophylactic local therapy.

There are two major challenges in studying prophylactic local therapy in patients with metastatic disease. First, local complications often represent the initial manifestation of de novo metastatic disease, precluding prevention outside of a robust screening program. Second, patients with de novo metastatic disease may experience a competing risk of death that varies by tumor type, disease biology, metastatic burden, and available systemic therapies. This could limit the window during which prophylactic local therapy could reduce morbidity. Therefore, to inform clinical trial design, estimates of complications due to local progression must account for events that occur too early for a prophylactic intervention to be effective as well as for competing mortality risks.

In this study, we characterize the cumulative incidence of SPOREs in de novo metastatic cancer, excluding events that are presenting symptoms and accounting for death as a competing risk. In order to capture the impact of varying degrees of metastatic virulence, we focus on de novo metastatic disease in three common cancer types with distinct natural histories: prostate cancer (3-year OS ∼60%) [4], bladder cancer (3-year OS ∼30%) [5], and pancreatic cancer (1-year OS <5%) [6], while each carries the potential for clinically significant local organ-related morbidity. Our a priori hypothesis was that the incidence of SPOREs would be inversely related to overall prognosis. That is, prostate cancer patients would experience the highest long-term risks followed by bladder and then pancreatic cancers. These data may inform both future perspectives on and clinical trial design for prophylactic local interventions (i.e. radiotherapy) aimed at reducing organ-related morbidity.

## 2 Methods

### 2.1 Study design and population

We conducted a retrospective cohort study of 358 patients with de novo prostate, bladder, and pancreatic cancers managed at an academic tertiary care center in the United States between January 1st, 2010, and December 31st, 2023. Key inclusion criteria were age 18 or older with histological (preferred), laboratory (ie. Prostate specific antigen [PSA]), and/or radiographic diagnosis of de novo metastatic disease. Conventional imaging, including Computed Tomography (CT), Magnetic Resonance Imaging (MRI), and bone scan, were used in most cases, while functional imaging, including PET-based imaging such as Prostate-Specific Membrane Antigen Positron Emission Tomography (PSMA-PET) for prostate cancer when available, was also accepted for determination of metastatic disease. Key exclusion criteria included fewer than 3 documented encounters in the medical record, death within 1 month of diagnosis, and patients who received definitive local therapies upon diagnosis, such as surgery (ie. Whipple procedure), or definitive radiation to the pancreas, bladder, or prostate with non-palliative intent. Accordingly, patients with de novo oligometastatic disease managed with curative-intent treatment to the primary tumor were not included. This project was approved by the institutional review board.

### 2.2 Definition of a severe primary organ-related event

Definitions of severe primary organ- (prostate-, bladder-, and pancreas-) related events (SPOREs) are summarized in Table 1. The definition for prostate-related events was adapted from the PEACE-1 trial definition for serious genitourinary events, which is a widely accepted, clinically meaningful framework for identifying events arising from an intact primary prostate tumor. [7] The bladder-related event definition was similar to that of the prostate given a shared spectrum of obstructive and bleeding-related complications. Finally, there is no published, standardized definition for severe pancreas-related events, so its definition was developed based on input from multidisciplinary experts to capture the common clinically significant complications attributed to local tumor effects. The pancreas group was further split into pancreatic adenocarcinoma and other histologies (subsequently referred to as pancreatic ADENO) excluding neuroendocrine tumor (NET) histology and pancreatic NET only, due to significant differences in their disease biology and prognosis. [8] In addition to histology, the location of the primary pancreatic tumor (head and body) was also evaluated separately.

**Table 1:** Definition of severe primary organ- (pancreas-, bladder-, and prostate-) related events (SPOREs).

Pancreas-related event	Definition	Prostate/bladder-related event	Definition
***Severe abdominal pain***	Requires palliative radiation, nerve block, invasive intervention for pain, **or** IV pain medication for ≥3 consecutive days	***Urinary catheter placement***	Placement of a Foley catheter for obstruction, urinary retention, or bladder decompression. Not as a part of routine/planned procedure
***Severe malnutrition***	Feeding tube placement (DHT, G-tube, J-tube) **or** initiation of TPN due to severe malnutrition	***Double-J ureteral stent placement***	Retrograde ureteral stent placement to relieve ureteral obstruction or hydronephrosis
***Gastrointestinal bleeding***	Requires IR embolization, EGD with cauterization, or palliative radiation for hemostasis	***Nephrostomy tube placement***	Percutaneous nephrostomy tube placement for upper urinary tract obstruction or urinary diversion
***Biliary obstruction / cholangitis***	Clinically diagnosed biliary obstruction or cholangitis requiring intervention	***Transurethral resection of the prostate (TURP)/Transurethral bladder tumor resection (TURBT)***	Endoscopic resection of prostatic tissue for obstruction or retention; typically, palliative in advanced disease. Not as a part of initial work up
***Gastrointestinal obstruction***	NG tube placement, emergent surgical decompression, duodenal stenting, or bypass surgery	***Palliative radiation***	Prostate/bladder-directed radiotherapy delivered outside routine indications for symptom control
***Acute pancreatitis***	Clinically diagnosed acute pancreatitis with associated complications	***Radical prostatectomy/cystectomy***	Surgical removal of the prostate/bladder gland for local control or symptom management in rare cases

### 2.3 Cumulative incidence of organ-related event and censoring

Patients were followed until the occurrence of a SPORE, death, or last known clinical encounter. Patients who did not experience an event were censored at the date of the last documented follow-up. Because death precludes the subsequent development of a SPORE, death was treated as a competing risk in our analyses, and cumulative incidence functions were estimated accordingly. SPOREs occurring within 30 days of diagnosis were excluded to restrict the analysis to patients potentially eligible for prophylactic local therapy and to avoid misclassifying presenting symptoms as preventable outcomes.

### 2.4 Statistical analysis

Baseline clinical characteristics were summarized using descriptive statistics. Cumulative incidence functions (CIFs) were generated for each cancer type and each location of the primary pancreatic tumor using the Fine-Gray sub-distribution framework, with death treated as a competing event instead of a censoring event because death precludes subsequent observation of a SPORE. CIF estimates were reported at various timepoints (6, 12, 24, and 60 months) and compared across cancer types and primary pancreatic tumor locations using Gray's test. Overall survival (OS) was defined as the time from diagnosis to death from any cause. Patients alive at last follow-up or lost to follow up were censored. OS was estimated using the Kaplan–Meier method and compared between groups (type and location) using the log-rank test. All analyses were performed in R (Version 2024.12.1 + 563) using the *cmprsk* and *survival* packages.

## 3 Results

### 3.1 Cohort characteristics

This study of de novo metastatic disease included 104 patients with prostate cancer, 59 patients with de novo metastatic bladder cancer, 195 patients with pancreatic cancer (including both ADENO and NET as well as body and head tumors). The pancreatic ADENO group included 172 adenocarcinoma cases and 6 rare other histologies because these subtypes were too few for separate analysis. Baseline characteristics were summarized in Table 2. The cohorts were heterogeneous with respect to demographic composition, performance status, metastatic distribution, histology, and first-line systemic therapy. Prostate cancer patients were treated with ADT-based systemic therapies, with pretreatment PSA and Gleason score reported only for this cohort. Bladder cancer patients received cisplatin-containing or non-cisplatin-containing systemic therapy, whereas patients with pancreatic cancer had distinct systemic treatment patterns based on histology, performance status, comorbidities, clinical trial eligibility, and evolving standards of care. The median follow-up time was 60.4, 40.9, 57.2, and 69.4 months for the prostate, bladder, pancreatic ADENO, and pancreatic NET cohort, respectively.

**Table 2:** Baseline demographic and clinical characteristics of patients with de novo metastatic prostate, bladder, and pancreatic (both ADENO and NET). Values are shown as n (%) unless otherwise indicated.

Characteristic	Prostate	Bladder	Pancreatic (NET and ADENO§)
Age at Diagnosis†	69 (49–89)	71 (32–87)	67 (37–87)
Sex	Male: 104 (100%)	Male: 40 (68%) Female: 19 (32%)	Male: 98 (50%) Female: 97 (50%)
Race	White: 62 (60%) Black: 37 (36%) Asian: 1 (1%) Other: 4 (4%)	White: 40 (68%) Black: 12 (20%) Asian: 3 (5%) Other: 4 (7%)	White: 168 (86%) Black: 14 (7%) Asian: 3 (2%) Other: 10 (5%)
Smoking History	Yes: 56 (54%) No: 48 (46%)	Yes: 40 (68%) No: 19 (32%)	Yes: 87 (45%) No: 108 (55%)
Histologic Type	Adenocarcinoma: 100 (96%) Small cell NET: 4 (4%)	Urothelial/Transitional cell carcinoma: 51 (86%) Small cell NET: 5 (9%) Other⁎: 3 (5%)	Adenocarcinoma: 172 (88%) Neuroendocrine tumor: 17 (9%) Other⁎⁎⁎: 6 (3%)
Tumor Location	–	–	Head: 97 (50%) Body⁎⁎⁎⁎: 98 (50%)
ECOG	0: 50 (48%) 1: 36 (35%) 2: 10 (10%) 3: 5 (5%) Unknown: 3 (3%)	0: 17 (29%) 1: 18 (31%) 2: 8 (14%) 3: 13 (22%) Unknown: 3 (5%)	0: 68 (35%) 1: 97 (50%) 2: 24 (12%) 3: 2 (1%) Unknown: 4 (2%)
Metastasis Site	Both: 60 (57%) Visceral/bone mets only: 40 (39%) Lymph node mets only: 4 (4%)	Both: 35 (59%) Visceral/bone mets only: 17 (29%) Lymph node mets only: 7 (12%)	Both: 102 (52%) Visceral/bone mets only: 93 (48%) Lymph node mets only: 0 (0%)
First-Line Systemic Therapy	Androgen deprivation therapy ± other systemic therapies	Included cisplatin? Yes: 20 (34%) No: 39 (66%)	(m)FOLFIRINOX: 49 (25%) FOLFOX: 19 (10%) Gem/Abraxane: 87 (45%) Gem alone: 13 (7%) Other⁎⁎⁎⁎⁎: 23 (12%) None: 4 (2%)
Pre-treatment PSA (ng/mL)	≤10: 10 (10%) >10–≤50: 26 (25%) >50–≤100: 11 (11%) >100: 54 (52%) Unknown: 3 (3%)	–	–
Gleason Score	7: 8 (8%) 8: 15 (14%) 9: 32 (31%) 10: 10 (10%) Unknown: 39 (38%)	–	–
Type of Organ-Related Event	Total: 11 (11%) Nephrostomy tube: 4 (36%) Foley catheter⁎⁎: 7 (64%)	Total: 15 (25%) Nephrostomy tube: 10 (67%) Foley catheter⁎⁎: 2 (13%) Palliative RT: 3 (20%)	Total: 79 (41%) Abdominal pain: 21 (27%) Biliary obstruction/cholangitis: 31 (39%) GI obstruction: 14 (18%) GI bleed: 3 (4%) Malnutrition: 10 (13%)

**§** The pancreatic ADENO group included 172 adenocarcinoma cases and 6 rare other histologies because these subtypes were too few for separate analysis.

† Data are median (range).

⁎ Other histologic types include sarcomatoid carcinoma (1 patients), adenocarcinoma (1), and squamous cell carcinoma (1).

⁎⁎ Foley catheter placement for urinary obstruction, not done as a part of routine procedures.

⁎⁎⁎ Other histologic types include atypical carcinoid tumor (3), mixed carcinoid adenocarcinoma (2), and intraductal papillary mucinous carcinoma (1).

⁎⁎⁎⁎ Charts were reviewed sequentially from a predefined patient list until the target sample size was reached; therefore, pancreatic tumor location could be classified only as head or body, and tail tumors were not analyzed separately.

⁎⁎⁎⁎⁎ Other regimens include capecitabine, temozolomide, somatostatin analogs, platinum-based agents, nivolumab (as a part of clinic trials), etoposide, everolimus, or some combination of these therapies.

### 3.2 Cumulative incidence

**Fig. 1:**
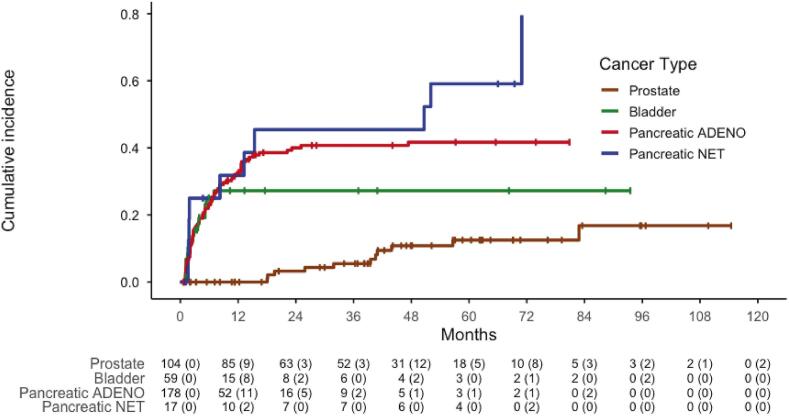
Cumulative incidence of SPOREs by cancer type with death treated as a competing event. Tick marks indicate censoring due to loss to follow-up. Numbers at risk (with censored patients in parentheses) are shown below. The cumulative incidence of SPOREs differed significantly across cancer types (Gray's test *p* < 0.001).

**Fig. 2:**
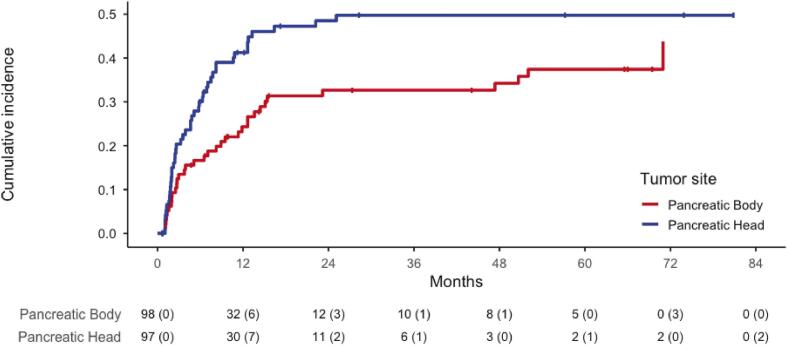
Cumulative incidence of SPOREs for pancreatic tumors by primary tumor location (body vs head), with death treated as a competing event. Tick marks indicate censoring due to loss to follow-up. Numbers at risk (with censored patients in parentheses) are shown below. There was a trend toward higher cumulative incidence of SPOREs in pancreatic head tumors compared with body tumors (Gray's test *p* = 0.054).

At least 1 organ-related event was found in the 11/104 (11%) of the prostate, 15/59 (25%) of the bladder, 69/178 (39%) of the pancreatic ADENO, 10/17 (59%) of the pancreatic NET, 34/98 (35%) of the pancreatic body, and 45/97 (46%) of the pancreatic head tumors. Based on the competing-risk function (Fig. 1), the cumulative incidence of SPOREs at 24 months was 3.3%, 27.2%, 40.0%, and 45.5% for prostate, bladder, pancreatic ADENO, and pancreatic NET respectively. Cumulative incidence of SPOREs plateaued after 24 months for bladder and pancreatic ADENO cancers, whereas the incidence increased to 12.5% for prostate cancer and 59.1% pancreatic NET at 60 months. For primary pancreatic tumor locations (Fig. 2), the cumulative incidence of SPOREs at 24 months were 48.5% and 32.7% for pancreatic head and pancreatic body tumors, respectively. The 6, 12, 24, and 60-month cumulative incidence of SPOREs are summarized in Table 3.

**Table 3:** Cumulative incidence of organ-related events by cancer type and pancreatic tumor location at 6, 12, 24, and 60 months. Values are cumulative incidence estimates with 95% confidence intervals.

Cumulative-Incidence Function (Deaths as Competing Events)	6 mo (%)	12 mo (%)	24 mo⁎ (%)	60 mo (%)
Bladder	25.2 (13.5–36.8)	27.2 (15.2–39.2)	27.2 (15.2–39.2)	27.2 (15.2–39.2)
Pancreatic ADENO	23.1 (16.8–29.4)	32.7 (25.6–39.8)	40.0 (32.5–47.5)	41.7 (34.0–49.4)
Pancreatic NET	25.0 (3.0–47.0)	31.8 (7.8–55.9)	45.5 (19.2–71.7)	59.1 (32.3–85.8)
Pancreatic head	30.1 (20.7–39.4)	41.2 (31.1–51.4)	48.5 (38.0–59.0)	49.8 (39.2–60.3)
Pancreatic body	16.6 (9.1–24.1)	24.3 (15.6–33.1)	32.7 (22.9–42.4)	37.4 (26.9–47.9)
Prostate	0.0 (0.0–0.0)	0.0 (0.0–0.0)	3.3 (0.0–6.9)	12.5 (5.1–19.9)

⁎ Censoring (lost to follow up) before 24 months was relatively uncommon and occurred in 16.9%, 9%,11.8%, 9.3%, 9.2%, and 11.5% of bladder, pancreatic ADENO, pancreatic NET patients, pancreatic head, pancreatic body, and prostate patients, respectively.

Gray's test demonstrated significant differences in the cumulative incidence of organ-related events across cancer cohorts (*p* < 0.001). The prostate cohort had the least cumulative incidence of severe primary organ-related events at any time point. The pancreatic ADENO and bladder cohort had a higher cumulative incidence of events (pancreatic ADENO > bladder) when compared to the prostate cohort, but most of the events occurred earlier on in the disease course. The pancreatic NET cohort, which only had 17 patients, had the highest incidence of events and these occurred both early and later in the disease course. In terms of pancreatic tumor location, pancreatic head tumors had a numerically higher cumulative incidence of SPOREs than body tumors with a trend toward statistical significance (Gray's test, *p* = 0.054).

### 3.3 SPORE type

In prostate cancer, foley catheter placement due to urinary obstruction is the most common event (64%), and 36% of events were nephrostomy tube placement. In bladder cancer, the most common type of event was nephrostomy tube placement (67%). Foley catheter placement due to urinary obstruction (13%) and palliative radiation (20%) were less common. In pancreatic cancer (all histologies and locations), biliary obstruction and/or cholangitis was the most common event (39%), followed by abdominal pain (27%), gastrointestinal obstruction (18%), failure to thrive or malnutrition (13%), and gastrointestinal bleed (4%).

### 3.4 Overall survival

**Fig. 3:**
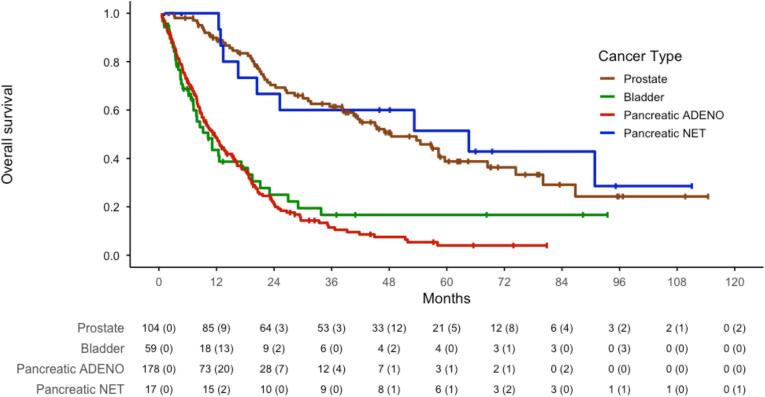
Overall survival by cancer type. Tick marks indicate censoring (patients alive at last follow-up or lost to follow-up). Numbers at risk (with censored patients in parentheses) are shown below. Overall survival differed significantly across cancer types (log-rank test p < 0.001).

**Fig. 4:**
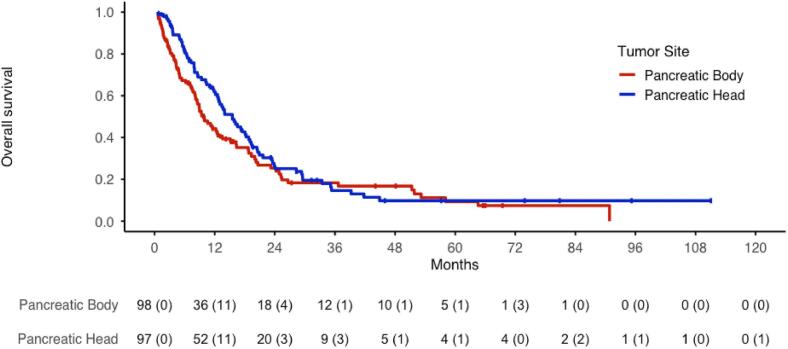
Overall survival by pancreatic tumor site (body vs head). Tick marks indicate censored observations. Numbers at risk (with censored patients in parentheses) are shown below. Overall survival did not differ significantly between tumor sites (log-rank test *p* = 0.167).

**Fig. 5:**
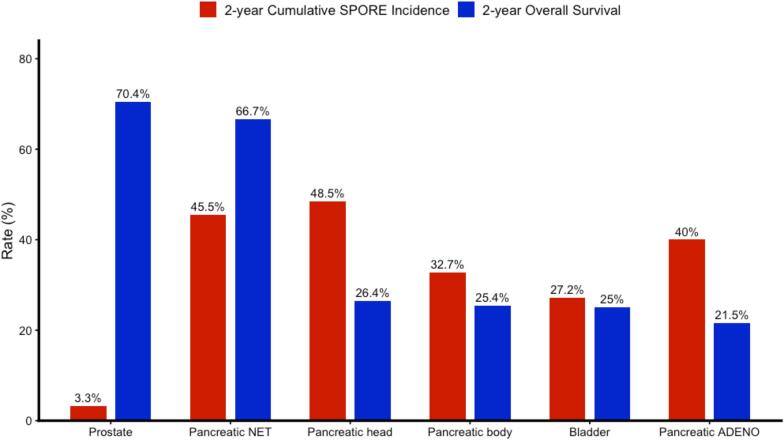
Comparison of 2-year cumulative incidence of severe primary organ-related events (SPOREs) and 2-year overall survival across cancer types and pancreatic tumor locations.

Median OS differed significantly across cancer types, with median OS of 48.4 months for prostate, 10.2 months for bladder, 11.5 months for pancreatic ADENO, and 64.6 months for pancreatic NET cohorts (Fig. 3, log-rank *p* < 0.001). Corresponding 2-year OS rates were 70.4%, 25.0%, 21.5%, and 66.7%, respectively. Among pancreatic cancers, median OS was 9.9 months for body tumors and 15.5 months for head tumors, without a statistically significant difference (Fig. 4, *p* = 0.167). The 2-year OS rates were similar between these two groups (25.4% vs 26.4%). To facilitate direct comparison between event risk and survival across disease groups and tumor locations, the 2-year cumulative incidence of SPOREs and 2-year overall survival are shown together in Fig. 5.

## 4 Discussion

Using the competing risk cumulative incidence analysis, we quantified the cumulative, type, and timing of SPOREs in de novo metastatic prostate, bladder, and pancreatic cancers. Because death precludes the subsequent development of a SPORE, competing-risk cumulative incidence estimates provide a more accurate assessment of event probability than Kaplan-Meier estimates, which treat deaths as censored. [9] These findings can guide future clinical trials by identifying cancer types with high SPORE rates, appropriate timing for intervention, and clinically meaningful endpoints that aim to improve patient's quality of life.

Our findings demonstrate that anatomic vulnerability, rather than metastatic virulence or overall prognosis, may be an important determinant of organ-specific morbidity. Firstly, pancreatic ADENOs and pancreatic NETs, despite differences in biological aggressiveness and overall prognosis, demonstrated similarly high cumulative incidence of SPOREs. In contrast, although pancreatic ADENOs and bladder cancers have similarly poor survival outcomes, pancreatic ADENOs has a significantly higher cumulative incidence of SPOREs. Additionally, pancreatic head tumors showed a trend toward higher cumulative incidence of SPOREs than body tumors despite similar overall survival, likely due to its proximity to the small bowel and biliary tree. This suggests that local anatomic susceptibility to mass-effect complications may outweigh disease aggressiveness in leading to organ-related morbidity.

Interestingly, despite prostate cancer developing from an organ traversed by a narrow urethra, bladder cancer exhibited both a higher and earlier incidence of SPOREs, predominantly driven by urinary obstruction. This finding is counterintuitive and may reflect differences in disease detection and biology. In the United States where prostate cancer screening is robust, patients presenting with de novo metastatic disease could represent a selected subgroup with more aggressive biology, whereas bladder cancer often presents with organ-related morbidities from having a modest local progression. This again supports local anatomic factors over metastatic burden in predicting organ-related morbidity. More importantly, prostate cancer's absolute incidence of SPOREs is relatively modest, yet randomized data demonstrate that prophylactic local therapy meaningfully reduced serious genitourinary events, as shown in the PEACE-1 trial [7]. In this context, the higher cumulative incidence of SPOREs observed in bladder and pancreatic cancers suggests that these disease sites may represent compelling candidates for prophylactic local strategies such as radiotherapy aimed at reducing morbidity.

Although overall survival is poor for pancreatic adenocarcinoma, the high incidence of early SPOREs suggests a clinically meaningful opportunity to reduce morbidity during this limited therapeutic window. Our competing-risk analysis accounts for early mortality, demonstrating that a substantial burden of organ-related morbidity remains despite the high competing risk of death.

There are several limitations to this study. This study has a retrospective design with reliance on chart documentation which may inaccurately estimate true event frequencies. However, in the PEACE-I trial, the incidence of serious genitourinary events at 5 years was approximately 30% in the standard of care without radiotherapy and with or without abiraterone arm and 10% in the standard of care plus radiotherapy with or without abiraterone arm. [7] Reassuringly, our retrospective assessment of severe primary prostate-related event incidence rate, approximately 15% at 5 years, is consistent with what was observed in the PEACE-I trial since our cohort includes patients treated with or without abiraterone (after its FDA approval in 2018) but no radiation to the primary. Additionally, two large population based-studies of pancreatic cancer have reported hospitalization rates from local tumor-related complications of approximately 40% (41% in Cardillo et al. and 43.5% in Sarkar et al.) [3], [10], findings that mirror the cumulative incidence of SPOREs observed in our combined pancreatic cohorts (41%) and provide external validation.

The study period spanned 2010–2023, during which systemic therapy standards evolved across all disease sites. Although available first-line systemic therapy data were summarized using broad disease-specific categories, regimen sequencing, treatment duration, response, clinical trial enrollment, and subsequent lines of therapy were not uniformly available. In addition, treatment selection was influenced by performance status, comorbidity, disease biology, and evolving standards of care, thus limiting the interpretability of treatment-era comparisons. Therefore, we are unable to fully account for systemic therapy heterogeneity when interpreting SPOREs and survival outcomes.

Other drawbacks include the definitions of SPORE, except for prostate cancer, are not validated from prior major clinical trials which may introduce heterogeneity and bias. Lastly, we performed exploratory logistic regression to evaluate predictors of organ-related events, but the small number of events and small overall sample size resulted in unstable estimates and no meaningful associations. Thus, we did not include these results. Furthermore, as a single-institution study, the relative distribution of cancer types reflects the available study population rather than the prevalence of metastatic cancers encountered in routine oncology practice.

In conclusion, using a competing-risk framework, we demonstrated that SPOREs represent a quantifiable and clinically relevant burden across de novo metastatic pancreatic, bladder, and prostate cancer. Anatomic vulnerability rather than metastatic virulence appears to be an important factor for a given cancer type. These data provide a foundation for designing prospective trials of prophylactic local therapy aimed at reducing organ-specific morbidity and improving patient-centered outcomes.

## Declaration of generative AI and AI-assisted technologies in the manuscript preparation process

During the preparation of this work the author(s) used ChatGPT for grammar editing and improving clarity of the manuscript. After using this tool/service, the author(s) reviewed and edited the content as needed and take(s) full responsibility for the content of the published article**.**

## Data sharing statement

Deidentified individual participant data underlying the results reported in this article will be made available upon reasonable request to the corresponding author, following approval of a proposal and execution of a data use agreement, and in accordance with institutional review board approval and applicable privacy regulations.

## CRediT authorship contribution statement

**Hefei Liu:** Conceptualization, Methodology, Data curation, Formal analysis, Investigation, Visualization, Writing – original draft. **Dafna Merhav:** Data curation, Investigation. **Naia Tabakovic:** Data curation, Investigation. **Ronac Mamtani:** Methodology, Supervision, Writing – review & editing. **Karishma Khullar:** Methodology, Supervision, Writing – review & editing. **John Christodouleas:** Conceptualization, Methodology, Supervision, Writing – review & editing.

## Funding statement

This research did not receive any specific grant from funding agencies in the public, commercial, or not-for-profit sectors.

## Declaration of competing interest

The authors declare the following financial interests/personal relationships which may be considered as potential competing interests: Dr. Hefei Liu reports prior employment at Varian Medical Systems more than 36 months ago and received recent consulting fees from Elekta, now currently an employee at Elekta. Ms. Dafna Merhav and Ms. Naia Merhav have nothing to disclose. Dr. Ronac Mamtani has nothing to disclose. Dr. Karishma Khullar has nothing to disclose. Dr. John Christodoules has a leadership role as the Head of Medical Affairs & Clinical Research at Elekta.
